# Survey of practices around the measurement and replacement of calcium in paediatric major trauma

**DOI:** 10.1136/bmjpo-2026-004588

**Published:** 2026-06-17

**Authors:** Owen Hibberd, Melanie Ranaweera, Spyridon Karageorgos, Kat Priddis, Ruud G Nijman, Andrew Tagg, Dani Hall, Damian Roland

**Affiliations:** 1 Emergency and Urgent Care Research in Cambridge (EURECA), PACE Section, Department of Medicine, Cambridge University, Cambridge, UK; 2 Blizard Institute, Queen Mary University of London, London, UK; 3 Department of Paediatric Emergency Medicine, University Hospital Southampton, Southampton, UK; 4 Paediatric Emergency Department, Aghia Sophia Children’s Hospital, Athens, Greece; 5 School of Medicine, University of Crete, Heraklion, Greece; 6 Children’s Emergency Department, West Hertfordshire Teaching Hospitals NHS Trust, Watford, UK; 7 Section of Pediatric Infectious Diseases, Imperial College of Science, Technology and Medicine, London, UK; 8 Western Health, Melbourne, Victoria, Australia; 9 Paediatric Academic Health Science Centre, Children’s Health Ireland, Dublin, Ireland; 10 SAPPHIRE Group, Population Health Sciences, Leicester University, Leicester, UK; 11 Paediatric Emergency Medicine Leicester Academic (PEMLA) Group, Children’s Emergency Department, Leicester Royal Infirmary, Leicester, UK

**Keywords:** Resuscitation, Physiology, Child Health

## Abstract

**Background:**

Ionised hypocalcaemia can worsen haemorrhage due to its role in clot formation, vascular tone and cardiac contractility. Current guidelines recommend maintaining normal ionised calcium levels in the bleeding trauma patient. However, guidance does not specify when to replace calcium, and for paediatric trauma, it is unclear when clinicians should measure or decide to replace calcium.

**Objectives:**

This study aimed to survey the practices, protocols and opinions regarding the measurement and replacement of calcium in paediatric major trauma.

**Methods:**

A cross-sectional survey with single-stage voluntary and snowball sampling. Individual emergency departments (EDs) responded to questions on departmental practices, while individual clinicians provided opinions on the measurement and replacement of calcium based on a clinical vignette with two variations (a haemodynamically stable and a haemodynamically unstable). The survey was administered using Online Surveys V3 (JISC, 2025). Respondents were invited to participate through collaborative research networks.

**Results:**

There were responses from 67 individual EDs and 99 individual respondents from 19 different countries. Most EDs had a paediatric major haemorrhage protocol (61/67, 91.0%), but this did not include calcium for 16/67 (23.9%) of the EDs. The timing of calcium replacement was either not included or not specified in most protocols (44/67, 65.7%), with variations in when it should be replaced when specified.

Opinions on considering calcium replacement before blood products were significantly different based on whether the patient in the vignette was clinically stable or unstable. Opinions on replacement doses of exogenous calcium were significantly different when the calcium was moderately low (<1.12 mmol/L), but were not significantly different if the calcium was severely low (<1.0 mmol/L).

**Conclusion:**

There is considerable variation among EDs regarding ionised calcium measurement and calcium replacement, both before blood product transfusion and in major haemorrhage protocols. Clinicians’ opinions on calcium measurement and replacement also vary widely. More research is needed to reach a consensus.

**Level of evidence:**

III.

**WHAT IS ALREADY KNOWN ON THIS TOPIC**

- Current guidelines recommend maintaining normal ionised calcium levels in the bleeding trauma patient, but do not specify when to replace calcium before receiving a transfusion, nor when to measure or replace as part of the major haemorrhage protocol.
- There is a lack of research exploring practice variation in how major haemorrhage protocols integrate ionised calcium measurement and calcium replacement for paediatric trauma.

**WHAT THIS STUDY ADDS**

- Most emergency departments surveyed could measure ionised calcium and had paediatric major haemorrhage protocol but the timing of calcium replacement was often unspecified or varied.
- Views on calcium replacement and doses before blood products varied based on the patient’s stability in the vignette; however, opinions on severe ionised hypocalcaemia (<1.0 mmol/L) were not significantly different, regardless of stability.

**HOW THIS STUDY MIGHT AFFECT RESEARCH, PRACTICE OR POLICY**

- This underscores the need for further research into optimal thresholds, timing, and integration of calcium into major haemorrhage protocols, as well as consensus on what defines clinically significant hypocalcaemia before blood product transfusion.

## Introduction

 Paediatric trauma remains a key cause of mortality globally, and many trauma-related deaths are caused by haemorrhage.^1^,^3^ If haemorrhage is promptly recognised and treated, it can be a potentially survivable cause of death.^1 2^ The classic ‘lethal triad’ of acidosis, coagulopathy and hypothermia interacts synergistically with each other, worsening haemorrhage and leading to a self-perpetuating cycle that is associated with increased mortality in trauma.^4 5^ The lethal triad has recently been reframed as the ‘diamond of death’ with integration of a fourth element, ionised hypocalcaemia.^5 6^ Both ionised hypocalcaemia and hypercalcaemia are thought to be detrimental in haemorrhage due to calcium’s role in clot formation, vascular tone and cardiac contractility.^4 6^ Transfusion of citrated blood products causes ionised hypocalcaemia due to calcium chelation with citrate, and this can be worsened in the presence of shock and reduced hepatic blood flow, leading to citrate accumulation.^7 8^ However, in trauma, early ionised hypocalcaemia can occur even without exogenous citrate, and the aetiology is complex, involving multiple potential mechanisms.^6^ Proposed mechanisms include, but are not limited to, direct calcium consumption and loss from coagulation and bleeding, haemodilution from the administration of intravenous fluids, and intracellular mechanisms shifts.^69^,^12^ Early ionised hypocalcaemia, occurring prior to blood product transfusion, has been reported in up to 50% of adult trauma patients and is associated with shock, coagulopathy, increased transfusion requirements and mortality.^11^,^13^ In paediatric trauma, the evidence is more limited and derived from a small number of heterogeneous studies, but similarly suggests associations with adverse outcomes, including the need for blood product transfusion, invasive interventions (such as interventional radiology or surgery) and poorer functional outcomes.^914^,^17^ Thus, it may be important to measure calcium in trauma patients, regardless of whether they have received a blood transfusion. Currently, there is no guidance on when clinicians should measure ionised calcium prior to blood transfusion in patients who may develop early ionised hypocalcaemia before haemorrhage is formally identified.

Current guidelines recommend maintaining normal calcium levels in the bleeding trauma patient.^18 19^ However, guidance does not specify when to measure or replace calcium before a transfusion, when the patient deteriorates, or as part of the major haemorrhage protocol, thereby risking both undertreatment of ionised hypocalcaemia and inappropriate replacement, which may lead to iatrogenic ionised hypercalcaemia.^18 19^ Consequently, there is wide variation in practice among major haemorrhage protocols, and clinicians have different opinions on the timings of when calcium should be measured and replaced.^20 21^ For paediatric trauma, it is therefore unclear when clinicians should measure calcium levels or decide to replace calcium, depending on local major haemorrhage protocols and their professional judgement.

This study surveyed the practices and opinions around the measurement and replacement of calcium in paediatric major trauma. In particular, the survey aimed to examine variation among emergency departments (EDs) in calcium measurement and replacement, which are incorporated into major haemorrhage protocols to provide contextual insight.

## Methods

### Study design

This was a cross-sectional survey with a voluntary convenience sample using snowball dissemination.^22^ The survey consisted of 21 questions, split across three main sections (online supplemental appendix 1).

### Survey sections

In the first section, survey questions measured the respondent’s role and expertise, while also providing contextual information on their hospital and trauma care provision. In this section, a senior doctor is defined as a general practitioner/family doctor, consultant, attending physician or equivalent, provided that all training and certification have been completed. A major trauma centre (MTC)/MTC equivalent was defined as a specialist hospital that has resources available 24 hours a day to manage severely injured patients, while a trauma unit (TU)/TU equivalent was defined as a hospital responsible for the local management of less severe injuries and the stabilisation and transfer of more severely injured patients and a local emergency hospital provides basic trauma care for those who do not need the input of a TU or MTC.^23^

The second section explored the practices and protocols for different aspects of calcium measurement and protocolised placement within major haemorrhage protocols.

Third and finally, there was a clinical vignette with two variations (a haemodynamically stable polytrauma and a haemodynamically unstable polytrauma) that explored opinions on managing trauma cases, with a focus on the measurement and replacement of calcium. The vignette was designed to explore clinician decision-making regarding calcium measurement and replacement prior to blood product administration or confirmation of major haemorrhage, rather than the activation of a major haemorrhage protocol. Haemodynamic variations in the vignette were included as a key factor that might influence early calcium measurement and replacement, particularly when occult bleeding is suspected.

### Feasibility

We demonstrated the feasibility of obtaining valid and reliable information from these questions in a previous study in the prehospital environment.^20^ Before distributing the questionnaire, colleagues from the authors’ institution who represented the target population pretested the survey.

### Eligibility

Any clinician treating paediatric major trauma patients in their practice was eligible to respond to the survey. The first question of the survey was used to determine eligibility, and it recorded whether a respondent was excluded because they did not treat paediatric major trauma patients in their clinical practice. All questions were mandatory, and respondents could not progress through the survey or submit their response without completing all questions, which prevented incomplete responses.

### Survey administration

The survey aimed to include an international cohort of respondents to improve representation of the study population. The survey was administered using Online Surveys V3 (JISC, 2025). Voluntary respondents were invited to participate through the ‘Don’t Forget The Bubbles’ (DFTB), ‘Paediatric Emergency Research United Kingdom and Ireland’ (PERUKI) and ‘Research in European Paediatric Emergency Medicine’ (REPEM) networks. DFTB is an educational organisation focused on knowledge translation and accessibility for paediatric emergency medicine (PEM). The digital impact factor (which measures website authority and reach across multiple social media platforms) for DFTB is the highest worldwide in this area.^24^ PERUKI is the national PEM research network, comprising academics and clinicians across the UK and Ireland, while REPEM is a research network for PEM, comprising academics and clinicians across European countries. Although no countries were excluded, the survey was only available in English.

Before conducting the survey, an educational blog post outlining the equipoise in the current literature entitled ‘Calcium in trauma: What do and don’t we know’ was published on the DFTB platform.^25^ An infographic (online supplemental appendix 2) was also created to help facilitate the snowball sampling. The survey was launched on 23 June 2025 and advertised online on the DFTB platform through the blog and a web-based pop-up.^25^ The survey was also advertised on the PERUKI website (https://www.peruki.org) until the survey was closed on 12 September 2025. Awareness of the survey was also raised through emails distributed by DFTB on 17 July 2025, PERUKI on 25 June 2025 and 6 August 2025, and REPEM on 7 August 2025 and 5 September 2025.

The survey was divided into sections, with results from individual EDs required for questions related to practices and protocols, while individual respondents were needed for sections concerning opinions on the management of the clinical vignette. If multiple responses were received from the same individual ED, then these were identified and compared. If any discrepancies arose, the authors planned to contact the relevant ED clinical lead directly for further information, using publicly available online department contact details to clarify transfusion protocols. Since the individual respondents had provided information anonymously and could not be contacted directly, this approach was deemed necessary. Every response was assigned a unique response number to distinguish between multiple responses, even when answers were identical. However, no IP addresses or participant-identifiable information were recorded, and it was therefore not possible to determine if there were duplicate responses from individuals.

The study’s reporting followed the EQUATOR Network Consensus-Based Checklist for Reporting of Survey Studies (CROSS) checklist (online supplemental appendix 3).^26^

Although no formal patient or public involvement was involved in the design, conduct or reporting of this study, a wider multi-centre study being undertaken by the authors had previously established the importance of the topic with the Bristol Young Persons’ Advisory Group.^27 28^

### Statistical analysis

Results were reported descriptively as a number (percentage). For opinions on the measurement and replacement of calcium in haemodynamically stable and unstable paediatric major trauma patients, Fisher’s exact was used to compare categorical variables, given that some values were small.

Statistical significance was defined as p<0.05 and reported either as the value if insignificant or p<0.05 or <0.005 if significant. An exploratory multivariable logistic regression was used to further explore decision-making for dichotomous opinions in each vignette. To make opinions binary and suitable for the regression model, opinions of ‘no’ or ‘not sure’ were merged. Independent variables used were whether the country uses trauma networks, the hospital has an MTC/MTC equivalent, has a paediatric major haemorrhage protocol in the ED, and if the major haemorrhage protocol includes calcium. Logistic regression models were evaluated using Hosmer-Lemeshow goodness-of-fit. Analysis was performed using Prism V.10.6.0 (GraphPad Software, 2025).

No post-survey adjustments, such as imputation or weighting, were planned to address non-response bias. No sensitivity analysis was undertaken.

## Results

**Figure 1:**
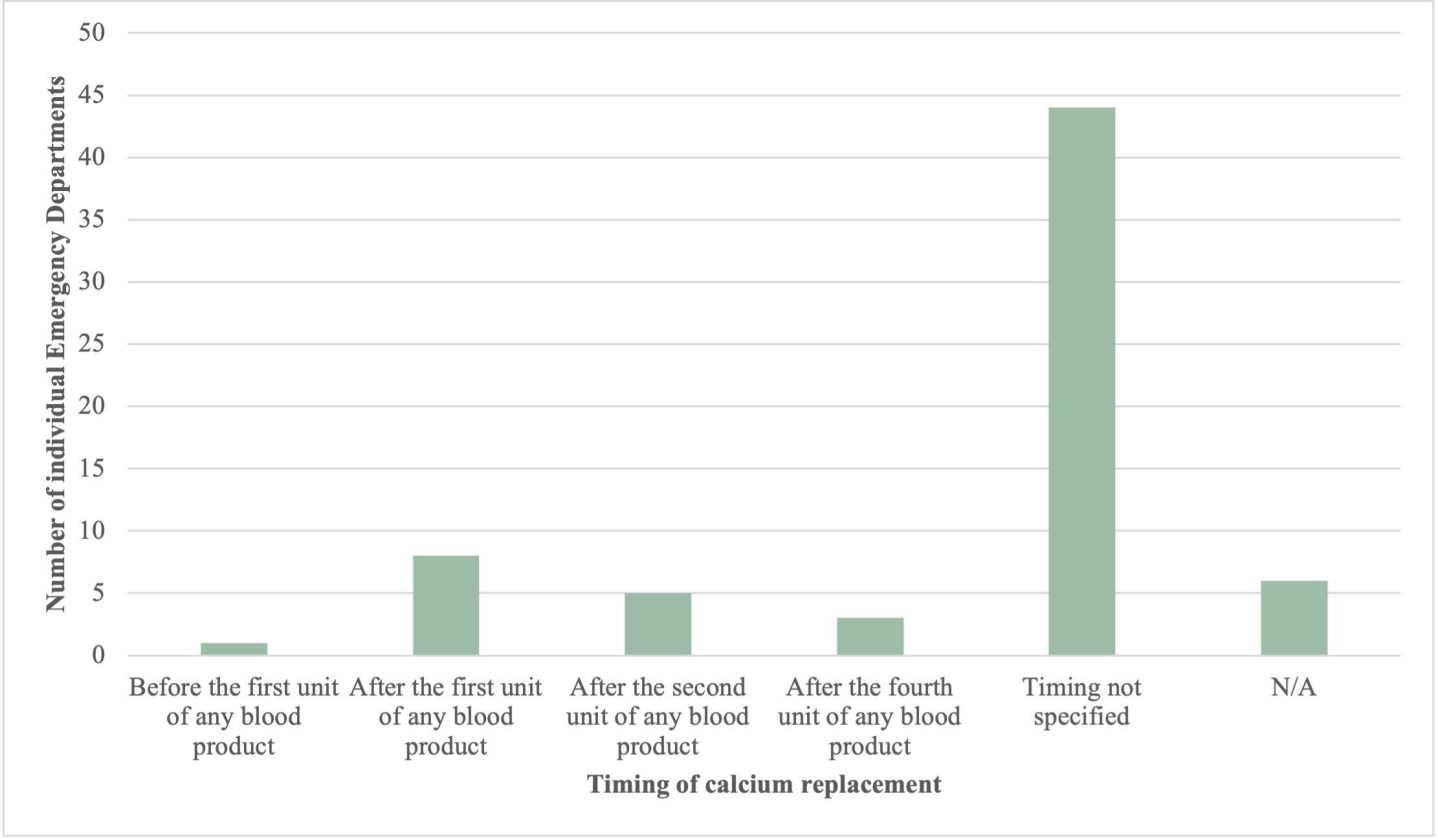
Paediatric major haemorrhage protocol recommendations on when to give the first calcium replacement (n=67).

There were responses from 67 individual EDs and 99 individual respondents. There were responses from 19 different countries, but most respondents were from the UK (65/99 (66%)). The maximum number of respondents from an individual ED was seven (figure 1).

There were no ineligible respondents based on the initial screening question. There were no incomplete responses and no discrepancies among respondents from the same ED which required further clarification. It was not possible to calculate the response rate due to the diverse and broad sampling technique, which collected a convenience sample.

### Practices and protocols

The individual EDs’ context for managing paediatric major trauma is shown in table 1. A detailed breakdown of results by international ED country is available in online supplemental appendix 4.

**Table 1:** Individual emergency departments’ context for managing paediatric major trauma (n=67)

Category	Response	Total, n=67 (%)	UK EDs, n=35 (%)	International EDs, n*=*32 (%)
Type of hospital	Not using trauma units/not applicable	2 (2.9)	0 (0.0)	2 (6.3)
	LEH/LEH equivalent	3 (4.5)	1 (2.9)	2 (6.3)
	TU/TU equivalent	20 (29.9)	17 (48.6)	3 (9.4)
	Paediatric MTC/MTC equivalent	17 (25.4)	5 (14.3)	12 (37.5)
	Combined paediatric/adult MTC/MTC equivalent	22 (32.8)	9 (25.7)	13 (40.7)
	Adult MTC/MTC equivalent	3 (4.5)	3 (8.5)	0 (0.0)
Does your emergency department use blood gas measurement which includes an ionised calcium?	Yes	56 (83.6)	34 (97.1)	22 (68.8)
	No	11 (16.4)	1 (2.9)	10 (31.2)
Does your emergency department use a standard panel of blood tests for major trauma patients (severely injured patients with an injury severity score of >12)	Yes	52 (77.6)	27 (77.1)	25 (78.1)
	No	15 (22.4)	8 (22.9)	7 (21.9)
If your emergency department uses a standard panel of blood tests for major trauma patients does this include an ionised calcium?	Yes	39 (58.2)	23 (65.7)	16 (50.0)
	No	8 (11.9)	5 (14.3)	13 (40.6)
	N/A	20 (29.9)	7 (20.0)	3 (9.4)
Does your emergency department have a paediatric major haemorrhage protocol?	Yes	61 (91.0)	35 (100.0)	26 (81.3)
	No	6 (9.0)	0 (0.0)	6 (18.7)
Does your paediatric major haemorrhage protocol include calcium replacement?	Yes	45 (67.2)	29 (82.9)	15 (46.9)
	No	17 (25.4)	6 (17.1)	11 (34.4)
	N/A	5 (7.4)	0 (0.0)	5 (15.6)

ED, emergency department; LEH, local emergency hospital; MTC, major trauma centre; TU, trauma unit.

There was a mixture of ED responses, with the highest proportion coming from hospitals with combined paediatric/adult MTC/MTC equivalent 22/67 (32.8%). Most EDs use blood gas measurement, which includes an ionised calcium 56/67 (83.6%), and this was most common in UK EDs (34/35 (97.1%). A standard panel of blood tests for major trauma patients was used in 52/67 (77.6%) EDs.

Most EDs had a paediatric major haemorrhage protocol (61/67, 91.0%). All UK EDs (35/35, 100%) had a paediatric major haemorrhage protocol and of these 29/35 (82.9%) mentioned calcium. For all EDs, major haemorrhage protocols did not include calcium for 16/67 (23.9%) of the total EDs. The timing of calcium replacement was either not included or not specified in most protocols (44/67, 65.7%), with variations in when it should be replaced when specified (figure 1).

### Clinicians’ opinions

When exploring opinions on managing trauma cases, with a focus on the measurement and replacement of calcium, most individual respondents were senior doctors (72/99, 73%) who specialised in emergency medicine or PEM (88/99, 89%) (table 2).

**Table 2:** Individual respondents’ role and area of practice (n=99)

Category	Response	Number (%)
Role in the emergency department	Advanced care practitioner	1 (1)
	Nurse	1 (1)
	Physician assistant	0 (0)
	Recently qualified doctor (eg, FY1—ST3, junior resident)	4 (4)
	Middle-grade doctor (eg, ST3+, resident or senior resident)	21 (21)
	Senior doctor (eg, consultant, attending)	72 (73)
	Other	0 (0)
How long have you been qualified for?	0–2 years	4 (4)
	2–5 years	8 (8)
	5–10 years	25 (25)
	>10 years	62 (63)
What is your specialist area of practice?	Emergency medicine/paediatric emergency medicine	88 (89)
	Paediatrics	7 (7)
	Other	4 (4)

FY1, foundation year 1 (first year as a doctor); ST3+, specialist trainee 3 (at least 3 years of specialist training).

The opinions on the measurement and replacement of calcium in haemodynamically stable and unstable paediatric major trauma patients based on the clinical vignettes are shown in table 3.

**Table 3:** Opinions on the measurement and replacement of calcium in haemodynamically stable and unstable paediatric major trauma patients prior to blood product transfusion (n=99)

Category	Response	Stable vignette, number (%)	Unstable vignette, number (%)	P value
Would you consider measuring the ionised calcium on arrival?	Yes	71 (72)	86 (87)	<0.05
	No	21 (21)	8 (8)	
	Not sure	7 (7)	5 (5)	
Would you consider replacing the calcium if it was moderately low (<1.12 mmol/L)?	Yes	25 (25)	52 (53)	<0.005
	No	42 (42)	25 (25)	
	Would not measure calcium on arrival	12 (12)	6 (6)	
	Not sure	20 (20)	16 (16)	
If the calcium was moderately low (<1.12 mmol/L) what dose of calcium replacement would you give?	Full dose	18 (18)	34 (34)	<0.05
	Half dose	11 (11)	13 (13)	
	Would not give calcium prior to blood transfusion	44 (44)	31 (31)	
	Not sure	26 (26)	21 (21)	
Would you consider replacing the calcium if it was severely low (<1.0 mmol/L) on arrival?	Yes	68 (69)	83 (84)	<0.05
	No	5 (5)	3 (3)	
	Would not measure calcium on arrival	17 (17)	5 (5)	
	Not sure	9 (9)	8 (8)	
If the calcium was severely low (<1.0 mmol/L), what dose of calcium replacement would you give?	Full dose	58 (59)	71 (72)	0.1
	Half dose	7 (7)	6 (6)	
	Would not give calcium prior to blood transfusion	19 (19)	8 (8)	
	Not sure	15 (15)	14 (14)	

Most respondents would consider measuring ionised calcium on arrival, and this opinion was more likely in the haemodynamically unstable vignette (stable: 71/99 (72%) vs unstable: 86/99 (87%), p<0.05). Opinions on considering calcium replacement before blood products were significantly different based on whether the patient in the vignette was clinically stable or unstable. The most significant difference in opinions was seen when considering replacing calcium if it was moderately low (<1.12 mmol/L) (stable: 25/99 (25%) vs unstable: 52/99 (53%), p<0.005). Opinions on replacement doses of exogenous calcium were significantly different when the calcium was moderately low (<1.12 mmol/L), but were not significantly different if the calcium was severely low (<1.0 mmol/L) in the vignette.^911^,^13 18^

The multivariable logistic regression model (online supplemental appendix 5) examined the association between independent variables and clinical opinions in the vignette. There were no missing data for the variables included in the model. The multivariable logistic regression for opinions based on the stable patient vignette largely did not identify any significant determinants of decision-making, except for increased odds of measuring calcium on arrival if the country uses trauma networks, OR 4.6 (95% CI 1.2 to 20.1); full dose replacement if calcium was severely low (<1.0 mmol/L) and the ED had a paediatric major haemorrhage protocol, OR 2.5 (95% CI 1.0 to 6.5); and full dose replacement if calcium was moderately low (<1.12 mmol/L) and the hospital was an MTC/MTC equivalent, OR 13.4 (95% CI 1.9 to 369.8) (table 4).

**Table 4:** Multivariable logistic regression for clinicians’ opinions on the stable patient vignette

Independent variable	Stable polytrauma vignette				
	Would consider measuring calcium on arrival for stable polytrauma patient OR (95% CI)	Would consider replacing the calcium if it was severely low (<1.0 mmol/L) on arrival for a stable polytrauma patient OR (95% CI)	If the calcium was severely low (<1.0 mmol/L), would consider a full dose replacement for a stable polytrauma patient OR (95% CI)	Would consider replacing the calcium if it was moderately low (<1.12 mmol/L) for a stable polytrauma patient OR (95% CI)	If the calcium was moderately low (<1.12 mmol/L), would consider a full dose replacement for a stable polytrauma patient OR (95% CI)
Country uses trauma networks	**4.6 (1.2 to 20.1)**	0.9 (0.2 to 4.1)	0.8 (0.2 to 3.1)	0.7 (0.2 to 3.5)	7.1 (0.6 to 317.5)
Hospital is a major trauma centre/equivalent	1.02 (0.4 to 2.7)	1.1 (0.4 to 2.8)	1.8 (0.7 to 4.4)	1.6 (0.6 to 5.1)	**13.4 (1.9 to 369.8)**
Emergency department has a paediatric major haemorrhage protocol	2.2 (0.3 to 14)	0.5 (0.1 to 4.0)	0.3 (0.1 to 2.1)	0.2 (0.1 to 1.2)	0.1 (0.1 to 0.3)
Major haemorrhage protocol includes calcium	1.1 (0.3 to 3.2)	0.6 (0.2 to 1.6)	**2.5 (1.0 to 6.5)**	1.6 (0.5 to 5.5)	0.6 (0.2 to 2.4)

Bold font indicates a statistically significant result.

The multivariable logistic regression for opinions on the unstable patient vignette (table 5) largely did not identify any significant determinants of decision-making, except for respondents having increased odds of measuring the ionised calcium on arrival, with an OR of 8.1 (95% CI 1.1 to 68.3), and the use of a full dose for moderately low hypocalcaemia (<1.12 mmol/L) if the hospital was an MTC/MTC equivalent.

**Table 5:** Multivariable logistic regression for clinicians’ opinions on the unstable patient vignette

Independent variable	Unstable polytrauma vignette				
	Would consider measuring the ionised calcium on arrival for an unstable polytrauma patient OR (95% CI)	Would consider replacing the calcium if it was severely low (<1.0 mmol/L) on arrival for an unstable polytrauma patient OR (95% CI)	If the calcium was severely low (<1.0 mmol/L), would consider a full dose replacement for an unstable polytrauma patient OR (95% CI)	Would consider replacing the calcium if it was moderately low (<1.12 mmol/L) for an unstable polytrauma patient OR (95% CI)	If the calcium was moderately low (<1.12 mmol/L), would consider a full dose replacement for an unstable polytrauma patient OR (95% CI)
Country uses trauma networks	3.2 (0.6 to 15.5)	1.3 (0.2 to 6.4)	1.4 (0.3 to 5.7)	2.2 (0.5 to 11.4)	1.2 (0.3 to 7.1)
Hospital is a major trauma centre/equivalent	0.7 (0.1 to 2.9)	0.6 (0.2 to 2.1)	1.6 (0.6 to 4.2)	1.8 (0.8 to 4.5)	**3.0 (1.1 to 9.2)**
Emergency department has a paediatric major haemorrhage protocol	**8.1 (1.1 to 68.3)**	0.7 (0.1 to 5.8)	0.6 (0.1 to 3.5)	0.2 (0.1 to 1.4)	0.1 (0.1 to 0.8)
Major haemorrhage protocol includes calcium	1.1 (0.2 to 4.5)	1.1 (0.3 to 3.6)	2.2 (0.8 to 5.8)	0.6 (0.3 to 1.6)	1.7 (0.6 to 5.0)

Bold font indicates a statistically significant result.

67/99 (67.7%) of respondents expressed interest in participating in further research related to calcium disturbances in paediatric major trauma.

## Discussion

Most surveyed EDs could perform blood gas measurement of ionised calcium and had paediatric major haemorrhage protocols. However, the timing of calcium replacement was mostly unspecified or varied. The majority of respondents, mainly senior clinicians, considered measuring ionised calcium on arrival. Views on calcium replacement and doses before blood products varied based on the patient’s stability in the vignette; however, opinions on severe hypocalcaemia (<1.0 mmol/L) were not significantly different, regardless of stability.

### Blood tests

Blood gas measurement of ionised calcium can provide rapid and actionable results when managing paediatric trauma in the resuscitation room. This provides the ionised calcium (mmol/L), which is the unbound, freely active form of calcium and therefore clinically relevant.^6 29^ The blood gas also offers information on acid-base status, base deficit, and lactate levels, which may reflect perfusion and serve as predictors of coagulopathy.^30^ A standardised blood test panel used in trauma can also identify other relevant factors associated with coagulopathy and poor outcomes.^4 6 30 31^ Both the European guidelines on the management of major bleeding and coagulopathy following trauma and the National Institute for Health and Care Excellence major trauma guideline recommend urgent blood testing in trauma cases.^18 32^ These include blood gases, haemoglobin, blood lactate, base deficit and markers of coagulation such as prothrombin time/international normalised ratio, Clauss fibrinogen level and platelet count.^18 32^ Monitoring and maintaining ionised calcium levels within normal range following major trauma is also advised, especially during major haemorrhages.^18^ In this survey, nearly a quarter of EDs did not have a standard trauma panel, and just over half of these included ionised calcium measurement as part of their standard panel. This highlights variability in practice and presents a potential opportunity for further standardisation and research to inform future guidelines.

### Major haemorrhage protocols

Guidelines recommend using major haemorrhage protocols for both adults and children.^18 32^ Variability in major haemorrhage protocols is an established research priority and there is limited evidence exploring this currently.^33^ For example, a single-centre review in the USA of 98 paediatric major haemorrhage protocol activations across a heterogeneous population of paediatric patients, including trauma and non-trauma cases, demonstrated wide variability in transfusion volumes and component use.^34^ Although evidence of variability in major haemorrhage protocol utilisation exists—reflected in this survey—no prior studies have examined the variation in calcium replacement or described approaches to managing trauma-related hypocalcaemia before blood transfusion among paediatric major haemorrhage protocols. When this was explored in a survey among UK helicopter emergency medical services (HEMS) for all trauma patients, significant variation was observed regarding where calcium fits into various protocols, with no standardised approach.^20^ This is an area where further research is needed, given the evidence of harm from hypercalcaemia and the risk that protocolised replacement may under-treat some patients while over-treating others, emphasising the importance of monitoring calcium levels.^11 35^ Some protocols may also incorporate calcium as a physiological target rather than provide explicit replacement guidance; as such, further research could also benefit from assessing precision trauma care, with guided treatments based on the patient’s calcium level.

### Clinicians’ opinions

Opinions on the measurement and replacement of calcium were mainly provided by senior clinicians in this survey. Several CIs were wide, which likely reflects the small sample size. Although this cohort is not fully representative of all clinicians who treat paediatric trauma, they do reflect real-life clinical practice, where it is likely that senior clinicians lead the management of paediatric trauma. Even with such an experienced group, many responses still indicated ‘not sure’. The survey did not assess whether calcium gluconate or calcium chloride was used. However, while this represents a potential source of unmeasured practice variation, existing guidance and evidence generally favour calcium chloride.^6 18^ A 10 mL dose of 10% calcium chloride contains approximately 270 mg of elemental calcium, compared with 90 mg in 10 mL of 10% calcium gluconate.^6 18^ In addition, calcium gluconate requires hepatic metabolism to release ionised calcium, which may delay its effect and be less reliable in the context of haemorrhagic shock.^6 18^ A recent survey of all UK HEMS also examined clinicians’ opinions on calcium replacement in trauma, with the research lead or lead clinician from each service responding.^20^ The UK HEMS survey found that clinicians vary significantly in their definitions of mild, moderate or severe ionised hypocalcaemia, highlighting that clear thresholds for what constitutes significant levels are not universally agreed on.^20^ There was also considerable variation in opinions on administering calcium before any blood products and the doses clinicians would use.^20^ However, similar to this survey, a majority opinion was that in severe ionised hypocalcaemia (levels not defined in the UK HEMS survey), a full dose of replacement would be preferred.^20^ The results from these surveys are valuable for hypothesis generation and for demonstrating variability in opinions. Future studies should aim to define significance levels before conducting trials on replacement. Steps should be taken towards reaching consensus on safe and effective calcium replacement, whether through a standardised protocol or by moving towards patient-centred measurement and replacement based on biochemistry.

### Limitations

As with any survey using voluntary snowball sampling, there is a risk of response bias, and the response rate could not be calculated, which may further introduce bias. Moreover, the population reached via research networks is more likely to be familiar with research, and the pre-survey educational blog may have primed respondents and influenced perceived uncertainty, which is a potential limitation that warrants acknowledgement. There was also clustering from individual centres, with seven responses from one ED. Nevertheless, it was advertised across multiple platforms to target a representative group of EDs and individuals, with responses coming from various countries and EDs, improving the generalisability of the results. This did not allow the response rate to be calculated, but it had the advantage of enabling a specific audience to be targeted. However, despite advertising, lower- and middle-income countries were underrepresented, and the results may lack external validity in this setting. Similarly, providing the survey exclusively in the English language is likely to have limited the number of international responses. Moreover, restricting submissions to only complete responses may have limited the total number of responses, but it has enhanced the overall data quality.

The exploratory multivariable logistic regression provided valuable insights into clinicians’ opinions; this only included four variables, and there is the possibility of additional confounders. For example, the clinicians’ country could not be included in the model due to the low number of responses from other countries relative to the UK, which may be a significant confounder. Given the small sample size, regression findings should be interpreted cautiously.

The vignettes did not explicitly confirm haemorrhage, which may have led to variable interpretation of them; however, this may reflect variation in clinician interpretation of early trauma physiology rather than protocol-driven care. There may also be unaccounted-for variation in reference ranges for ionised calcium and in definitions of full- and half-dose calcium replacement between EDs, which could have influenced the results or been biased by the wording ‘severely low’ and ‘moderately low’. However, in the absence of international consensus on thresholds and dosing, this was mitigated by adopting definitions consistent with existing literature. As such, this study provides important foundational work to inform future research aimed at establishing consensus. In addition to this, the survey did not explore whether protocols included physiological calcium targets, potentially underestimating the extent to which calcium is incorporated into major haemorrhage pathways. Similarly, the survey did not explore what constitutes a standard guideline or standard trauma panel, or whether EDs were using viscoelastic measurements. However, it was considered that this would require a substantial amount of information from respondents, which could have increased the risk of survey fatigue and errors in results.

## Conclusion

Although most EDs could measure calcium and had paediatric major haemorrhage protocols, the timing of calcium replacement was often unspecified or varied. Views on calcium measurement, replacement and dosage in a patient who had not received a blood transfusion also varied widely. This underscores the need for further research into what constitutes a safe and effective major haemorrhage protocol, as well as consensus on what defines clinically significant hypocalcaemia before blood product transfusion.

## Supplementary material

10.1136/bmjpo-2026-004588 online supplemental file 1

10.1136/bmjpo-2026-004588 online supplemental file 2

10.1136/bmjpo-2026-004588 online supplemental file 3

10.1136/bmjpo-2026-004588 online supplemental file 4

10.1136/bmjpo-2026-004588 online supplemental file 5

## Data Availability

Data are available upon reasonable request.
