# Supplementary material for: Survey of practices around the measurement and replacement of calcium in paediatric major trauma
Source: BMJ Paediatr Open. 2026 Jun 17;10(1):e004588. doi: 10.1136/bmjpo-2026-004588 (PMC13289376; doi:10.1136/bmjpo-2026-004588)
Supplement: online supplemental file 1 [file bmjpo-10-1-s001.pdf]

A survey of current practices and opinions around the measurement and replacement of calcium in paediatric trauma

Introduction

The *Don't Forget The Bubbles* team would like to thank you for participating in this survey and contributing to paediatric research.

We know that low ionised calcium levels might be harmful in paediatric trauma. However, there is no consensus on when to measure or replace calcium, resulting in highly variable practice. This survey hopes to develop new knowledge by exploring the current practices for measuring and replacing calcium in paediatric trauma.

We are looking to include all clinicians, nurses, and allied health professionals who treat children and work in the emergency department.

The survey is split into three sections, estimated to take 10 minutes in total to complete. The first section lets us know about your role, hospital and practice. The other sections are related to the practice and protocols in your work area. We then have a clinical vignette exploring your opinions on managing trauma cases with a focus on the measurement and replacement of calcium. **We recommend that you have a copy of your massive transfusion protocol to hand to help you answer some of the questions.**

At the end of the survey, you can access a link to download a certificate recognising your support for and involvement in research. Collaboration is essential in research, and we hope with your help, we can enhance our understanding of this area and improve our knowledge and treatment of injured children.

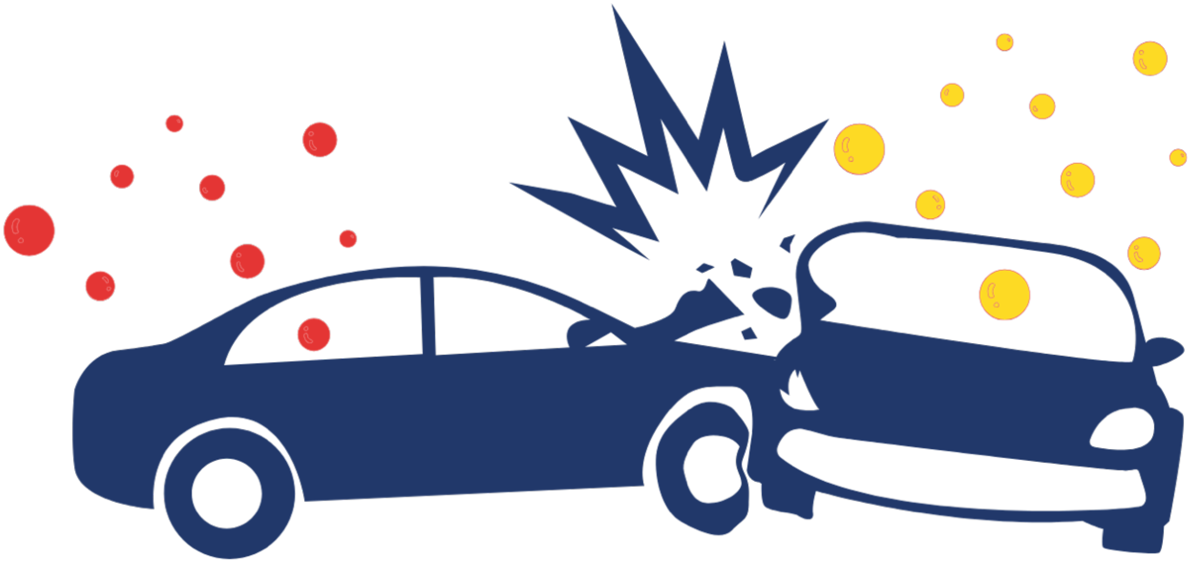

Eligibility Questions

1. **Does your scope of practice involve treating severely injury children and adolescents in the emergency department? ***

- ◯ Yes
- ◯ No

Demographics

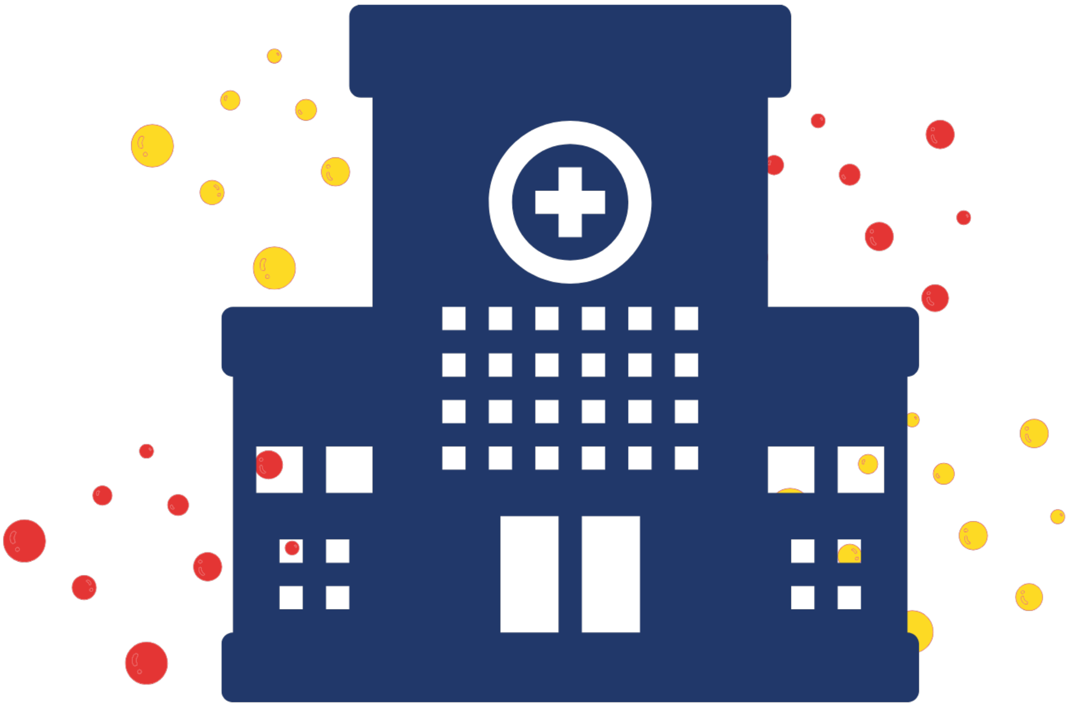

2. **What is your role e.g. Advanced Care Practitioner, Doctor, Nurse, Physician Assistant?** *

- ◯ Advanced Care Practitioner
- ◯ Nurse
- ◯ Physician Assistant
- ◯ Recently qualified doctor (e.g. FY1 - ST3, junior resident)
- ◯ Middle-grade doctor (e.g. ST3+, resident or senior resident)
- ◯ Senior doctor (e.g. GP, consultant, attending)
- ◯ Other

3. **How long have you been qualified for?** *

- ◯ Not yet qualified

- 0 - 2 years
- 2 - 5 years
- 5 - 10 years
- >10 years

4. **What is your specialist area of practice?** *

- Emergency Medicine/Paediatric Emergency Medicine
- Paediatrics
- Paediatric Surgery
- Other

5. **What hospital do you currently work at?** *

0/32,000 characters

6. **In which country is your emergency department?** *

0/32,000 characters

7. **Does your country utilise trauma networks?** *

- Yes
- No

8. **If your hospital utilises trauma networks, is your hospital a Major Trauma Centre (MTC)/ MTC equivalent (a specialist hospital that has resources available 24 hours a day to manage severely injured patients) or a Trauma Unit (TU)/TU equivalent (a hospital responsible for the local management of less severe injuries and the stabilisation and transfer of more severely injured patients)?** *

- MTC/MTC equivalent

- ◯ TU/TU equivalent
- ◯ Neither an MTC/TU
- ◯ Not applicable

9. **If your hospital is a MTC/MTC equivalent, is it a standalone paediatric centre, a combined paediatric/adult centre, or an adult centre?** *

- ◯ Paediatric MTC/MTC equivalent
- ◯ Combined paediatric/adult MTC/MTC equivalent
- ◯ Adult MTC/MTC equivalent
- ◯ Not applicable

Practice and protocols

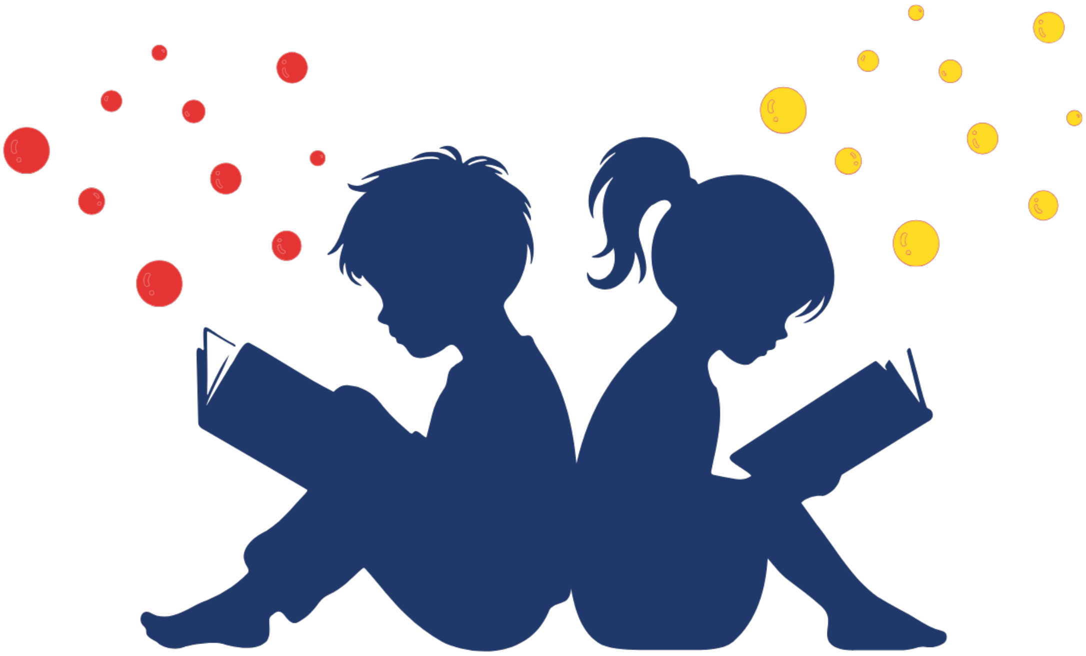

10. **Does your emergency department use point-of-care blood gas measurement which includes an ionised calcium? ***

◯ Yes
◯ No

11. **Does your emergency department use a standard panel of blood tests for major trauma patients (severely injured patients with an injury severity score of >12) ***

◯ Yes
◯ No

12. **If your emergency department uses a standard panel of blood tests for major trauma patients does this include an ionised calcium? ***

◯ Yes
◯ No

- ◯ Not applicable

13. **Does your emergency department have a paediatric massive transfusion protocol?** *

- ◯ Yes
- ◯ No

14. **Does your paediatric massive transfusion protocol include calcium replacement?** *

- ◯ Yes
- ◯ No
- ◯ Not applicable

15. **When does your paediatric massive transfusion protocol recommend giving the first calcium replacement?** *

- ◯ Before the first unit of any blood product
- ◯ After the first unit of any blood product
- ◯ After the second unit of any blood product
- ◯ After the third unit of any blood product
- ◯ After the fourth unit of any blood product
- ◯ Timing not specified
- ◯ Not applicable

16. **Does your emergency department have any guidance on what to do if the calcium is low before a blood transfusion?** *

- ◯ Yes
- ◯ No

17. **If your emergency department has guidance on what to do if the calcium level is low before a blood transfusion, what does it say?** *

0/32,000 characters

Opinions and clinical vignette

In this section, we will explore your opinions and hypothetical measurement and management of low calcium levels in paediatric trauma.

We present a hypothetical case with two variations on the trauma theme. All of these patients are hypothetically seen on arrival and none of them have received any blood products. All vital signs parameters are based on APLS values.

Thank you!

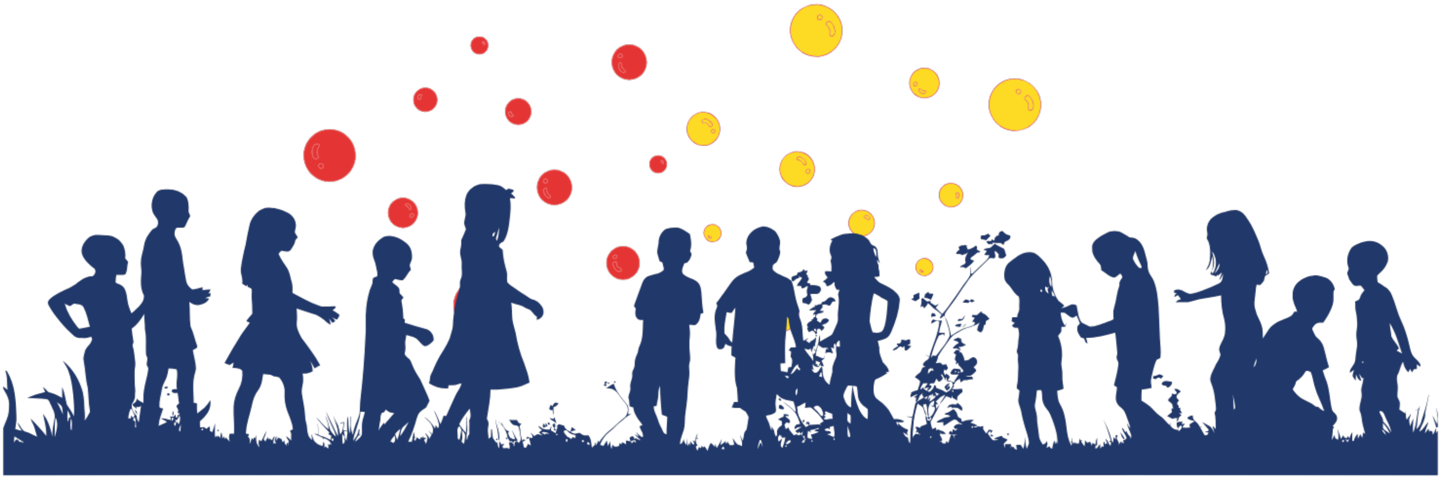

Case 1. Frodo (A)

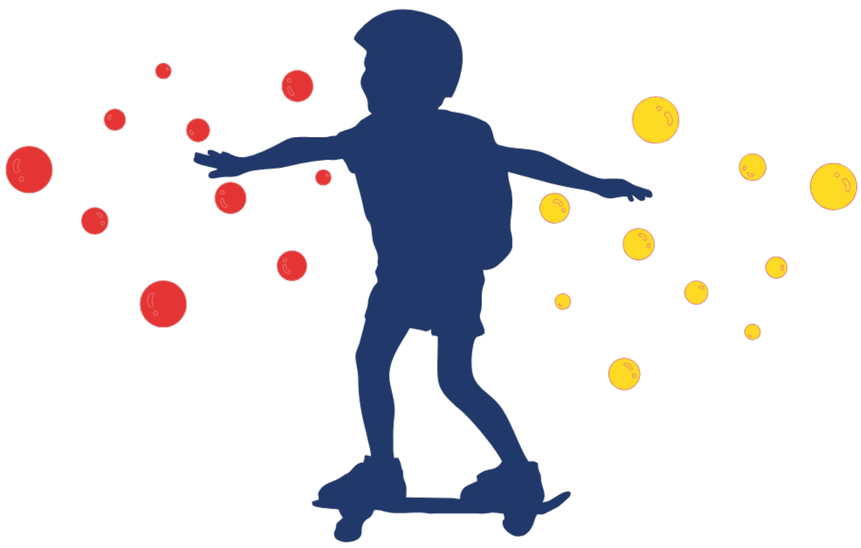

Patient Details
Frodo.
Eight years old.
Skateboarding and hit by a car.

Injury Details
Multiple injuries. Contusions to the head, chest wall tenderness and bruising, abdominal bruising and guarding in the right upper quadrant.

Vital Signs
Heart rate: 75 [70-110]
Systolic blood pressure: 100 [86-118]
Respiratory rate: 20 [18-22]
Temperature: 37.0 [36.4-37.5]
Glasgow Coma Score: 15 [15]

18. **Would you consider measuring the ionised calcium on arrival?** *

- ◯ Yes
- ◯ No
- ◯ Not sure

19. **Would you consider replacing the calcium if it was severely low (<1.0 mmol/L) on arrival?** *

- ◯ Yes
- ◯ No
- ◯ Would not measure calcium on arrival
- ◯ Not sure

20. **If the calcium was severely low (<1.0 mmol/L), what dose of calcium replacement would you give?** *

- ◯ Full dose
- ◯ Half dose
- ◯ Would not give calcium prior to blood transfusion
- ◯ Not sure

21. **Would you consider replacing the calcium if it was moderately low (<1.12mmol/L)?** *

- ◯ Yes
- ◯ No
- ◯ Would not measure calcium on arrival
- ◯ Not sure

22. **If the calcium was moderately low (<1.12 mmol/L) what dose of calcium replacement would you give?** *

- ◯ Full dose
- ◯ Half dose
- ◯ Would not give calcium prior to blood transfusion
- ◯ Not sure

Case 1. Frodo (B)

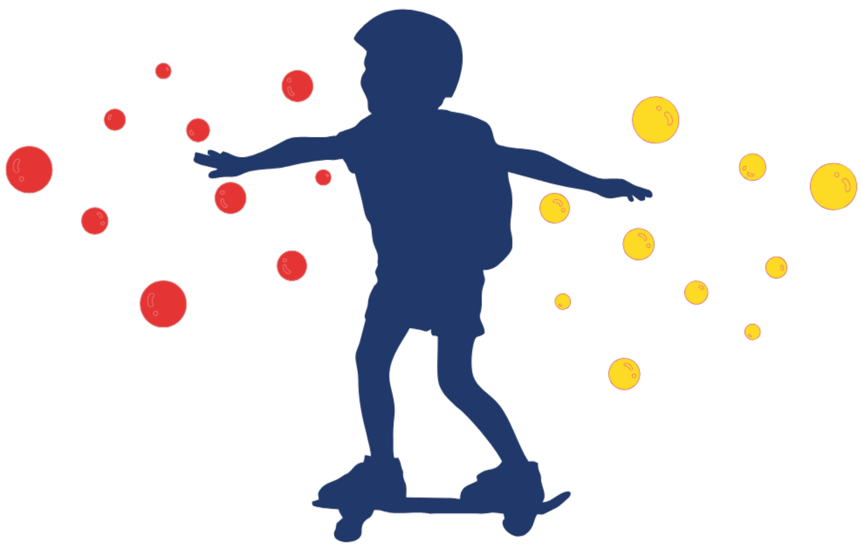

Patient Details
Frodo.
Eight years old.
Skateboarding and hit by a car.

Injury Details
Multiple injuries. Contusions to the head, chest wall tenderness and bruising, abdominal bruising and guarding in the right upper quadrant.

Vital Signs
Heart rate: **140** [70-110]
Systolic blood pressure: **80** [86-118]
Respiratory rate: **26** [18-22]
Temperature: 36.7 [36.4-37.4]
Glasgow Coma Score: 15 [15]

23. **Would you consider measuring the ionised calcium on arrival?** *

- ◯ Yes
- ◯ No
- ◯ Not sure

24. **Would you consider replacing the calcium if it was severely low (<1.0 mmol/L) on arrival? ***

- ◯ Yes
- ◯ No
- ◯ Would not measure calcium on arrival
- ◯ Not sure

25. **If the calcium was severely low (<1.0 mmol/L), what dose of calcium replacement would you give? ***

- ◯ Full dose
- ◯ Half dose
- ◯ Would not give calcium prior to blood transfusion
- ◯ Not sure

26. **Would you consider replacing the calcium if it was moderately low (<1.12mmol/L)? ***

- ◯ Yes
- ◯ No
- ◯ Would not measure calcium on arrival
- ◯ Not sure

27. **If the calcium was moderately low (<1.12 mmol/L) what dose of calcium replacement would you give? ***

- ◯ Full dose
- ◯ Half dose
- ◯ Would not give calcium prior to blood transfusion
- ◯ Not sure

Thank you!

28. **Would you be interested in being involved in further hypocalcaemia research?** *

- ◯ Yes
- ◯ No

Please click here to download a certificate. We have left it so that you can add your name to this so that the survey remains anonymous.

We would be grateful if you could share this survey with your friends and colleagues.

Thank you for your time!
