# Supplementary figures and images for: Survey of practices around the measurement and replacement of calcium in paediatric major trauma
Source: BMJ Paediatr Open. 2026 Jun 17;10(1):e004588. doi: 10.1136/bmjpo-2026-004588 (PMC13289376; doi:10.1136/bmjpo-2026-004588)

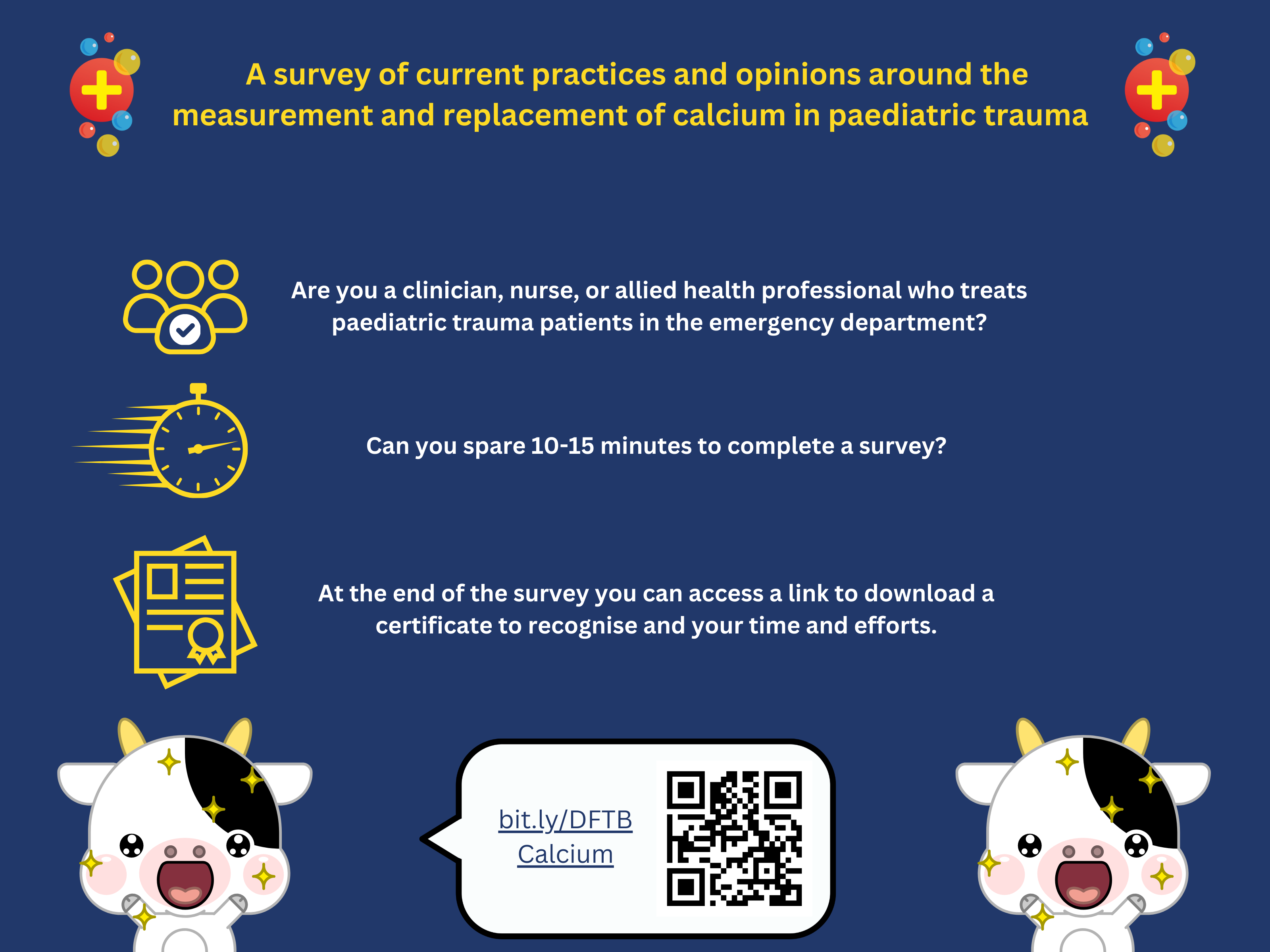

Supplement: online supplemental file 2 [file bmjpo-10-1-s002.png]
