# Supplementary material for: Survey of practices around the measurement and replacement of calcium in paediatric major trauma
Source: BMJ Paediatr Open. 2026 Jun 17;10(1):e004588. doi: 10.1136/bmjpo-2026-004588 (PMC13289376; doi:10.1136/bmjpo-2026-004588)
Supplement: Supplementary file 6 [file bmjpo-2026-004588.reviewer_comments.pdf]

PEER REVIEW HISTORY

BMJ Paediatrics Open publishes all reviews undertaken for accepted manuscripts. Reviewers are asked to complete a checklist review form and are provided with free text boxes to elaborate on their assessment. These free text comments are reproduced below.

ARTICLE DETAILS

**TITLE (PROVISIONAL)**	A survey of practices around the measurement and replacement of calcium in paediatric major trauma
**AUTHORS**	Hibberd, Owen<br>Ranaweera, Melanie<br>Karageorgos, Spyridon<br>Priddis, Katherine Louisa<br>Nijman, Ruud<br>Tagg, Andrew<br>Hall, Dani<br>Roland, Damian

VERSION 1 – REVIEW

**REVIEWER NAME**	George, Shane
**REVIEWER AFFILIATION**	
**REVIEWER CONFLICT OF INTEREST**	No Conflict of Interest
**AI DISCLOSURE**	No, I have not used AI
**DATE REVIEW RETURNED**	27-Feb-2026

**GENERAL COMMENTS**	Thank you for the opportunity to review this manuscript on an important topic in paediatric haemorrhage.<br><br>My first recommendation is one of terminology, the manuscript currently talks about calcium replacement in “massive transfusion”, but what I think the authors are referring to is calcium replacement in “Major Haemorrhage” or “Critical Bleeding”. There has been an intentional shift away from the terminology of “Massive Transfusion Protocols” in favour of “Major Haemorrhage Protocols” or “Critical Bleeding Protocols” – the reason for this change is based on recognising that haemorrhage is the problem that needs to be stopped, and transfusion is ONE of the interventions that are used to help stop the haemorrhage. Calcium is indeed another one of those tools.<br><br>Many critical bleeding pathways include physiological targets, with calcium being one of those targets (for example: https://www.blood.gov.au/module-6-neonatal-and-paediatrics-patient-blood-management-guidelines#critical-bleeding-protocol-template) . The survey did not appear to ask about this being present, only if there was guidance on replacement. This is a potential source of bias in the reporting and should be addressed.<br><br>The questions posed in the Clinical vignette are somewhat confusing. It is unclear to me why haemodynamically unstable vs stable was chosen for this question. The Vignettes do not describe any major haemorrhage, while a concealed intrabdominal injury is inferred, it is not clear to me how these patients would activate the

	major haemorrhage protocol and/or if the patient needed a blood transfusion. It is confusing that the paper concentrates on MHPs, yet seems to be asking the question about whether clinicians would measure and replace Ca++ BEFORE they activate the MHP or give blood products. Could you please clarify the intent of these cases and the relevance to MHPs?<br><br>In the case with abnormal vital signs while it is likely that this patient would receive some fluid +/- blood, there needed to be specific information to confirm that the signs were due to haemorrhage (e.g. positive eFAST scan and persistent hypotension despite 20ml/kg crystalloid given pre-hospital). The lack of case definition creates the risk of different "imagined" patients for respondents which may be the cause of variation in responses rather than true practice variation.<br><br>Multivariable regression on such small sample size is problematic and should at best be referred to as exploratory. I also question the validity of combining "no" and "unsure" responses – these are not the same thing and "unsure" could equally be grouped with "yes" the combination in the negative creates bias.<br><br>While there is no doubt that DFTB is a great vehicle to develop interest in a survey and distribute to a broad audience, the publication of a blog post explaining equipoise introduces the risk of priming respondents and inflating perceived uncertainty in their responses.<br><br>Overall the paper provides an interesting insight into the variation is paediatric critical bleeding management, however the combination of both self-reported (hypothetical) clinician practice and clinical practice guidelines into a single paper complicates the delivery.

**REVIEWER NAME**	Mullen, Stephen
**REVIEWER AFFILIATION**	
**REVIEWER CONFLICT OF INTEREST**	No Conflict of Interest
**AI DISCLOSURE**	I used Claude to formulate the layout of my review. It was not used to review the paper itself or to change any content in my view.
**DATE REVIEW RETURNED**	24-Mar-2026

**GENERAL COMMENTS**	General<br>Good paper on a relevant topic. However, the paper feels out of balance — it is focused on calcium (Ca) in paediatric trauma yet repeatedly incorporates massive haemorrhage protocol (MHP) content to a degree not warranted by the presented results (in my view). The introduction and discussion require considerable reworking to reflect this. The core finding — that when Ca is low, most clinicians feel it should be replaced, with some variation — is not new information, but remains relevant as a foundation for future studies.<br><br>Title & Aims<br>The title is specific to Ca but the aims include variation in MHP. One of these needs to change. There is insufficient data presented to support detailed discussion of MHP variation (in my opinion).

	Introduction<br>• The first paragraph should be more focused on Ca given the paper's scope.<br>• Consider including any available data on the mortality burden of the "lethal diamond," including adult studies if paediatric data are limited.<br>• It would be helpful to clarify whether one objective is to highlight that Ca is sometimes replaced as part of MHP even when POCT levels are within the normal range — if so, this should be made explicit, along with the potential benefits and concerns of Ca replacement.<br>• MHP is referenced extensively but its relevance is not clearly explained. If it relates to the point above, this should be made explicit.<br>• Consider addressing the options for timing and method of Ca measurement (e.g. pre- or post-transfusion, on ED arrival, when clinical deterioration occurs, as part of routine bloods).<br><br>Methods<br>• The response rate cannot be calculated — acknowledged by the authors, but this deserves greater emphasis in the discussion regarding its impact on the findings.<br>• Voluntary snowball sampling via the named organisations is likely to over-represent clinicians already engaged in research networks, introducing potential bias. This should be more explicitly addressed.<br>• Please clarify what is meant by "single-stage voluntary and snowballing sample" — has this methodology been used in published literature? If so, please reference.<br>• "Questionnaire instruments" is unclear — please rephrase.<br>• A senior clinician is defined as a general practitioner; for many readers this means a family doctor. Please clarify the intended definition.<br>• The study design section would benefit from sub-headings for readability.<br>• All questions are described as mandatory to prevent missing data, yet the results section refers to missing data being reported descriptively. Please clarify this inconsistency.<br>• Please reference the definitions used for low and moderately low Ca values in the vignettes, confirm consistency with international standards, and clarify how variation in measurements between regions/countries was accounted for.<br><br>Results<br>• Seven respondents from a single ED represents considerable bias and should be acknowledged.<br>• Figure 1: axes need labelling.<br>• Half-dose is mentioned in the results but not defined in the methods. Please define full and half dose, and address any international variation.<br>• Confirm whether "severe" and "moderately low" reflect the exact wording used in the vignettes — these terms could introduce bias.<br>• Please clarify whether the form of Ca used (gluconate vs. chloride) was explored.<br>• Several confidence intervals are wide and should be discussed.<br><br>Discussion<br>• The paragraph beginning with POCT is lengthy and contains material not directly relevant to this study.<br>• The paragraph on MHP feels out of place given the presented

	data.<br>• The limitations section requires expansion to address the methodological concerns raised above.<br>• The conclusion states "considerable variation amongst EDs regarding the role" — it is unclear whether this refers to variation in ED protocols or in ED physician opinion on when to replace Ca. Please clarify.<br><br>What Is Already Known<br>• First statement: please clarify whether the data on Ca disturbances and link to mortality refers to adult or paediatric populations. "Disturbance" is vague — please specify (hypocalcaemia, hypercalcaemia, or both).<br>• Third point regarding MHPs: the relevance to this paper is unclear in its current form. A more specific reference to Ca within MHP would improve clarity.<br><br>What This Study Adds<br>• The statement that all EDs can measure Ca assumes lab testing — clarify if point-of-care testing (POCT) is intended.<br><br>Future Research<br>• The reference to MHP should be more explicitly linked to Ca to improve relevance and clarity.

VERSION 1 – AUTHOR RESPONSE

Response to Reviewers

Thank you to both reviewers for their valuable and insightful comments. We believe these have helped improve the clarity, scope, and focus of the study. By refining the study's focus, clarifying terminology, strengthening the methodological description, and expanding the discussion of limitations, we believe these revisions have significantly enhanced the overall quality and interpretability of the manuscript. We are sincerely grateful for all comments received and are happy to make any further changes as required to improve the study.

Reviewer 1: Dr Shane George

Thank you for the opportunity to review this manuscript on an important topic in paediatric haemorrhage.

- My first recommendation is one of terminology, the manuscript currently talks about calcium replacement in "massive transfusion", but what I think the authors are referring to is calcium

replacement in “Major Haemorrhage” or “Critical Bleeding”. There has been an intentional shift away from the terminology of “Massive Transfusion Protocols” in favour of “Major Haemorrhage Protocols” or “Critical Bleeding Protocols” – the reason for this change is based on recognising that haemorrhage is the problem that needs to be stopped, and transfusion is ONE of the interventions that are used to help stop the haemorrhage. Calcium is indeed another one of those tools.

  - **Thank you. This is an excellent point. We have updated this to major haemorrhage and major haemorrhage protocols throughout.**

- Many critical bleeding pathways include physiological targets, with calcium being one of those targets (for example: https://www.blood.gov.au/module-6-neonatal-and-paediatrics-patient-blood-management-guidelines#critical-bleeding-protocol-template) . The survey did not appear to ask about this being present, only if there was guidance on replacement. This is a potential source of bias in the reporting and should be addressed.
  - **Thank you. We agree this is an excellent point. To reflect this, we have added the following to the discussion: ‘Some protocols may also incorporate calcium as a physiological target rather than provide explicit replacement guidance; as such, further research could also benefit from assessing precision trauma care, with guided treatments based on the patient's calcium level.’**
  - **We have also added the following to the limitations: ‘The survey did not explore whether protocols included physiological calcium targets, potentially underestimating the extent to which calcium is incorporated into major haemorrhage pathways.’**

- The questions posed in the Clinical vignette are somewhat confusing. It is unclear to me why haemodynamically unstable vs stable was chosen for this question. The Vignettes do not describe any major haemorrhage, while a concealed intrabdominal injury is inferred, it is not clear to me how these patients would activate the major haemorrhage protocol and/or if the patient needed a blood transfusion. It is confusing that the paper concentrates on MHPs, yet seems to be asking the question about whether clinicians would measure and replace Ca++

BEFORE they activate the MHP or give blood products. Could you please clarify the intent of these cases and the relevance to MHPs?

- **Thank you. We apologise that this was not clearer and are grateful to you for identifying this. The vignettes were designed to explore clinician decision-making before major haemorrhage is confirmed and before blood products are given, rather than after MHP activation itself. We chose haemodynamic stability versus instability because this is a clinically relevant factor that may influence early calcium measurement and replacement in paediatric trauma, particularly when occult bleeding is suspected. Our aim was to investigate practice variation in this early phase, as current guidance addresses calcium in bleeding trauma patients but does not clearly define when calcium should be measured or replaced before transfusion.**
- **In the introduction, we have added the sentence in red to clarify this: 'in trauma, early ionised hypocalcaemia can occur even without exogenous citrate, and the aetiology is complex, involving multiple potential mechanisms.[9] Proposed mechanisms include, but are not limited to, direct calcium consumption and loss from coagulation and bleeding, haemodilution from the administration of intravenous fluids, and intracellular mechanisms shifts.[9 13-16] Moreover, the classic 'lethal triad ' may further exacerbate or potentiate ionised hypocalcaemia.[9] Thus, it may be important to measure calcium in trauma patients, regardless of whether they have received a blood transfusion. Currently, there is no guidance on when clinicians should measure ionised calcium prior to blood transfusion in patients who may develop early ionised hypocalcaemia before haemorrhage is formally identified.'**
- **In the methods we have added: 'The vignette was designed to explore clinician decision-making regarding calcium measurement and replacement prior to blood product administration or confirmation of major haemorrhage, rather than the activation of a major haemorrhage protocol. Haemodynamic variations in the vignette were included as a key factor that might influence early calcium**

measurement and replacement, particularly when occult bleeding is suspected.'

- In the limitations we have added: 'The vignettes did not explicitly confirm haemorrhage, which may have led to variable interpretation of them; however, this may reflect variation in clinician interpretation of early trauma physiology rather than protocol-driven care.'

- In the case with abnormal vital signs while it is likely that this patient would receive some fluid +/- blood, there needed to be specific information to confirm that the signs were due to haemorrhage (e.g. positive eFAST scan and persistent hypotension despite 20ml/kg crystalloid given pre-hospital). The lack of case definition creates the risk of different "imagined" patients for respondents which may be the cause of variation in responses rather than true practice variation.
    - **Thank you. We hope we have addressed this with the updates above; however, if any further clarification is needed, we would be delighted to provide more information.**

- Multivariable regression on such small sample size is problematic and should at best be referred to as exploratory. I also question the validity of combining "no" and "unsure" responses – these are not the same thing and "unsure" could equally be grouped with "yes" the combination in the negative creates bias.
    - **Thank you. We appreciate the concern here. We have reframed the logistic regression as exploratory. Responses of 'no' and 'unsure' were combined to allow binary modelling and grouped as we felt 'unsure' was more likely to represent an action not being taken; however, we acknowledge these represent distinct constructs, and this may introduce bias. We hope the changes below have acknowledged this.**
    - **We have added the following to the methods: 'An exploratory multivariable logistic regression was used to further explore decision-making for dichotomous opinions in each vignette.'**

- **We have added the following to the limitations: ‘Given the small sample size, regression findings should be interpreted cautiously.’**

- While there is no doubt that DFTB is a great vehicle to develop interest in a survey and distribute to a broad audience, the publication of a blog post explaining equipoise introduces the risk of priming respondents and inflating perceived uncertainty in their responses.
  - **Thank you. We agree and have added the following to the limitations: ‘Moreover, the population reached via research networks is more likely to be familiar with research and the pre-survey educational blog may have primed respondents and influenced perceived uncertainty, which is a potential limitation needing acknowledgement.’**

- Overall the paper provides an interesting insight into the variation is paediatric critical bleeding management, however the combination of both self-reported (hypothetical) clinician practice and clinical practice guidelines into a single paper complicates the delivery.
  - **Thank you. We believe the topics are complementary and contextually informative of each other. We hope the reframing throughout this revision has improved clarity and flow, but we are open to reframing it as two separate studies if required.**

Reviewer 2: Dr Stephen Mullen

General

- Good paper on a relevant topic. However, the paper feels out of balance — it is focused on calcium (Ca) in paediatric trauma yet repeatedly incorporates massive haemorrhage protocol (MHP) content to a degree not warranted by the presented results (in my view). The introduction and discussion require considerable reworking to reflect this. The core finding — that when Ca is low, most clinicians feel it should be replaced, with some variation — is not new information, but remains relevant as a foundation for future studies.
    - **Thank you. We agree and feel that this paper is essential foundational work for future studies. We have tried to reduce the detail and the degree to which the major haemorrhage protocol is included and instead kept this when it is directly linked to calcium or provides contextual insight into the survey results.**

Title & Aims

- The title is specific to Ca but the aims include variation in MHP. One of these needs to change. There is insufficient data presented to support detailed discussion of MHP variation (in my opinion).
    - **Thank you. We have updated the aims to provide further clarity on the focus on calcium measurement. These now read as: 'This study surveyed the practices and opinions around the measurement and replacement of calcium in paediatric major trauma. In particular, the survey aimed to examine variation among Emergency Departments (EDs) in calcium measurement and replacement are incorporated into major haemorrhage protocols to provide contextual insight.'**

Introduction

- The first paragraph should be more focused on Ca given the paper's scope.
    - **Thank you. This has been revised to focus on calcium and provide further rationale for measurement prior to blood product transfusion.**

- **This now reads: ‘Paediatric trauma remains a key cause of mortality globally, and many trauma-related deaths are caused by haemorrhage.[1-3] If haemorrhage is promptly recognised and treated, it can be a potentially survivable cause of death.[1 2] The classic ‘lethal triad’ of acidosis, coagulopathy, and hypothermia interacts synergistically with each other, worsening haemorrhage and leading to a self-perpetuating cycle that is associated with increased mortality in trauma.[4 5]The lethal triad has recently been reframed as the ‘diamond of death’ with integration of a fourth element, ionised hypocalcaemia.[5 6] Both ionised hypo- and hypercalcaemia are thought to be detrimental in haemorrhage due to calcium’s role in clot formation, vascular tone, and cardiac contractility.[4 6] Transfusion of citrated blood products causes ionised hypocalcaemia due to calcium chelation with citrate, and this can be worsened in the presence of shock and reduced hepatic blood flow, leading to citrate accumulation.[7 8] However, in trauma, early ionised hypocalcaemia can occur even without exogenous citrate, and the aetiology is complex, involving multiple potential mechanisms.[6] Proposed mechanisms include, but are not limited to, direct calcium consumption and loss from coagulation and bleeding, haemodilution from the administration of intravenous fluids, and intracellular mechanisms shifts.[6 9-12] Early ionised hypocalcaemia, occurring prior to blood product transfusion, has been reported in up to 50% of adult trauma patients and is associated with shock, coagulopathy, increased transfusion requirements, and mortality.[11-13] In paediatric trauma, the evidence is more limited and derived from a small number of heterogeneous studies, but similarly suggests associations with adverse outcomes, including the need for blood product transfusion, invasive interventions (such as interventional radiology or surgery), and poorer functional outcomes.[9 14-17] Thus, it may be important to measure calcium in trauma patients, regardless of whether they have received a blood transfusion. Currently, there is no guidance on when clinicians should measure ionised calcium prior to blood transfusion in patients who may**

develop early ionised hypocalcaemia before haemorrhage is formally identified.'

- Consider including any available data on the mortality burden of the "lethal diamond," including adult studies if paediatric data are limited.
    - **Thank you. We have reframed this to clarify the mortality burden and mechanism and have provided references. We have avoided overexpanding this section, as a detailed discussion of trauma-induced coagulopathy would detract from the primary focus on calcium. Instead, we have strengthened the rationale by including additional background on early ionised hypocalcaemia prior to transfusion, and included paediatric studies and the two paediatric systematic reviews.**
    - **This now reads: 'The classic 'lethal triad' of acidosis, coagulopathy, and hypothermia interacts synergistically with each other, worsening haemorrhage and leading to a self-perpetuating cycle that is associated with increased mortality in trauma.[4] [5]The lethal triad has recently been reframed as the 'diamond of death' with integration of a fourth element, ionised hypocalcaemia.'**
    - **The section on early hypocalcaemia reads: 'Early ionised hypocalcaemia, occurring prior to blood product transfusion, has been reported in up to 50% of adult trauma patients and is associated with shock, coagulopathy, increased transfusion requirements, and mortality.[11-13] In paediatric trauma, the evidence is more limited and derived from a small number of heterogeneous studies, but similarly suggests associations with adverse outcomes, including the need for blood product transfusion, invasive interventions (such as interventional radiology or surgery), and poorer functional outcomes.'**

- It would be helpful to clarify whether one objective is to highlight that Ca is sometimes replaced as part of MHP even when POCT levels are within the normal range — if so, this should be made explicit, along with the potential benefits and concerns of Ca replacement.

- **Thank you. Although this is an excellent point, it was not one of the survey's explicit objectives. However, reflecting this, we have added the following to the introduction: 'However, guidance does not specify when to measure or replace calcium before receiving a transfusion, when the patient deteriorates, or as part of the major haemorrhage protocol, which risks both under-treatment of ionised hypocalcaemia and inappropriate replacement, which may lead to iatrogenic ionised hypercalcaemia'.**

- MHP is referenced extensively, but its relevance is not clearly explained. If it relates to the point above, this should be made explicit.
  - **Thank you. Overall, we have reduced discussion on major haemorrhage protocols and clarified the contextual link to calcium measurement and replacement. We hope we have also further explained the relevance.**

- Consider addressing the options for timing and method of Ca measurement (e.g. pre- or post-transfusion, on ED arrival, when clinical deterioration occurs, as part of routine bloods).
  - **Thank you. We have clarified the uncertainty surrounding the timing of calcium measurement and replacement. However, we feel the current evidence base is insufficient to provide detailed recommendations on specific timings (e.g. pre- or post-transfusion, on ED arrival, or during clinical deterioration). This lack of evidence further underscores the importance and relevance of our study.**
  - **This now reads as: 'However, guidance does not specify when to measure or replace calcium before receiving a transfusion, when the patient deteriorates, or as part of the major haemorrhage protocol, which risks both under-treatment of ionised hypocalcaemia and inappropriate replacement, which may lead to iatrogenic ionised hypercalcaemia.[12 13] Consequently, there is wide variation in practice amongst major haemorrhage protocols, and clinicians have different opinions on the timings of when calcium should be measured and replaced.[14 15]'**

Methods

- The response rate cannot be calculated — acknowledged by the authors, but this deserves greater emphasis in the discussion regarding its impact on the findings.
- Voluntary snowball sampling via the named organisations is likely to over-represent clinicians already engaged in research networks, introducing potential bias. This should be more explicitly addressed.
    - **Thank you. We believe we have addressed both of these points with the following addition in the limitations: 'As with any survey using voluntary snowball sampling, there is a risk of response bias, and the response rate could not be calculated, which may further introduce bias. Moreover, the population reached via research networks is more likely to be familiar with research, and the pre-survey educational blog may have primed respondents and influenced perceived uncertainty, which is a potential limitation that warrants acknowledgement.'**

- Please clarify what is meant by "single-stage voluntary and snowballing sample" — has this methodology been used in published literature? If so, please reference.
    - **Thank you, we have rephrased this and provided a reference for snowballing. This now reads: 'This was a cross-sectional survey with a voluntary convenience sample using snowball dissemination'.**
    - **Reference: Streeton R, Cooke M, Campbell J. Researching the researchers: using a snowballing technique. *Nurse Res* 2004;12(1):35-46. doi: 10.7748/nr2004.07.12.1.35.c5929**

- "Questionnaire instruments" is unclear — please rephrase.
    - **Thank you. This has been rephrased to: 'survey questions'.**

- A senior clinician is defined as a general practitioner; for many readers this means a family doctor. Please clarify the intended definition.

- **Thank you. We have updated this to: 'General Practitioner/Family Doctor'.**
- **We have kept this in the text as it was an option for clinicians to accept based upon the readership of *Don't Forget The Bubbles*. However, no General Practitioners/Family Doctors responded, so it may be that it is better to remove this from the text. We will be guided by your opinion here.**

- The study design section would benefit from sub-headings for readability.
  - **Thank you. The following sub-headings have been added: 'Study sections, Feasibility, and Eligibility'.**

- All questions are described as mandatory to prevent missing data, yet the results section refers to missing data being reported descriptively. Please clarify this inconsistency.
  - **Thank you. With apologies, as all questions were mandatory, there was no missing data, and this statement has now been removed.**

- Please reference the definitions used for low and moderately low Ca values in the vignettes, confirm consistency with international standards, and clarify how variation in measurements between regions/countries was accounted for.
  - **Thank you. References have been added to support the definitions of moderately low and severely low calcium used in the vignettes, drawing on a combination of existing guidance and published literature. However, there remains no clear international consensus on thresholds for moderate versus severe hypocalcaemia, which likely contributes to variation in practice.**
  - **The following has been added to the limitations: 'There may also be unaccounted-for variation in reference ranges for ionised calcium and in definitions of full- and half-dose calcium replacement between emergency departments, which could have influenced the results. However, in the absence of international consensus on thresholds and dosing, this was mitigated by adopting definitions consistent with existing literature. As such, this study**

provides important foundational work to inform future research aimed at establishing consensus.'

<u>Results</u>

- Seven respondents from a single ED represent considerable bias and should be acknowledged.
    - **Thank you. In the limitations, when discussing bias, we have added: 'There was also clustering from individual centres, with seven responses from one ED.'**

- Figure 1: axes need labelling.
    - **Thank you. This has been updated.**

- Half-dose is mentioned in the results but not defined in the methods. Please define full and half dose and address any international variation.
    - **Thank you. We have added the following to the limitations: 'There may also be unaccounted-for variation in reference ranges for ionised calcium and in definitions of full- and half-dose calcium replacement between emergency departments, which could have influenced the results or been biased by the wording "severely low" and "moderately low". However, in the absence of international consensus on thresholds and dosing, this was mitigated by adopting definitions consistent with existing literature. As such, this study provides important foundational work to inform future research aimed at establishing consensus.'**

- Confirm whether "severe" and "moderately low" reflect the exact wording used in the vignettes — these terms could introduce bias.
    - **Thank you. We agree and have added the following to the limitations: 'There may also be unaccounted-for variation in reference ranges for ionised calcium and in definitions of full- and half-dose calcium replacement between**

emergency departments, which could have influenced the results or been biased by the wording "severely low" and "moderately low".'

- Please clarify whether the form of Ca used (gluconate vs. chloride) was explored.
  - **Thank you. We recognise this may be of interest to readers and have added the following to the discussion: 'The survey did not assess whether calcium gluconate or calcium chloride was used. However, while this represents a potential source of unmeasured practice variation, existing guidance and evidence generally favour calcium chloride.[4 12] A 10 mL dose of 10% calcium chloride contains approximately 270 mg of elemental calcium, compared with 90 mg in 10 mL of 10% calcium gluconate.[4 12] In addition, calcium gluconate requires hepatic metabolism to release ionised calcium, which may delay its effect and be less reliable in the context of haemorrhagic shock.[4 12]'**

- Several confidence intervals are wide and should be discussed.
  - **Thank you. We have added the following to the discussion: 'Several confidence intervals were wide, which likely reflects the small sample size.'**

<u>**Discussion**</u>

- The paragraph beginning with POCT is lengthy and contains material not directly relevant to this study.
  - **Thank you. Sub-headings for the discussion section have been provided to help with readability (Blood tests, Major haemorrhage protocols, Clinicians' opinions).**
  - **We believe we have maintained a tighter focus on this section now, which reads: 'Blood gas measurement of ionised calcium can provide rapid and actionable results when managing paediatric trauma in the resuscitation room. This is provides the ionised calcium (mmol/L), which is the unbound, freely active form of calcium and therefore clinically relevant.[4 24] The blood gas also**

offers information on acid-base status, base deficit, and lactate levels, which may reflect perfusion and serve as predictors of coagulopathy.[25] A standardised blood test panel used in trauma can also identify other relevant factors associated with coagulopathy and poor outcomes.[4 5 25 26] Both the European guidelines on the management of major bleeding and coagulopathy following trauma and the National Institute for Health and Care Excellence (NICE) major trauma guideline recommend urgent blood testing in trauma cases.[12 27] These include blood gases, haemoglobin, blood lactate, base deficit, and markers of coagulation such as prothrombin time (PT)/international normalised ratio (INR), Clauss fibrinogen level, and platelet count.[12 27] Monitoring and maintaining ionised calcium levels within normal range following major trauma is also advised, especially during major haemorrhages.[12] In this survey, nearly a quarter of EDs did not have a standard trauma panel, and just over half of these included ionised calcium measurement as part of their standard panel. This highlights variability in practice and presents a potential opportunity for further standardisation and research to inform future guidelines.'

- The paragraph on MHP feels out of place given the presented data.
    - **Thank you. We agree that the discussion focused a bit too much on the evidence supporting major haemorrhage protocols in general and also included discussion on balanced transfusion ratios, which we have not explored in the data. As such, this has been shortened to focus on variability in major haemorrhage protocols and calcium's role.**
    - **This now reads: Guidelines recommend using major haemorrhage protocols for both adults and children.[12 27] Variability in major haemorrhage protocols is an established research priority and there is limited evidence exploring this currently.[28] For example, a single-centre review in the USA of 98 paediatric major haemorrhage protocol activations across a heterogeneous population of paediatric patients, including trauma and non-trauma cases, demonstrated**

wide variability in transfusion volumes and component use.[29] Although evidence of variability in major haemorrhage protocol utilisation exists—reflected in this survey—no prior studies have examined the variation in calcium replacement or described approaches to managing trauma-related hypocalcaemia before blood transfusion among paediatric major haemorrhage protocols. When this was explored in a survey among UK helicopter emergency medical services (HEMS) for all trauma patients, significant variation was observed regarding where calcium fits into various protocols, with no standardised approach.[14] This is an area where further research is needed, given the evidence of harm from hypercalcaemia and the risk that protocolised replacement may under-treat some patients while over-treating others, emphasising the importance of monitoring calcium levels.[10 30] Some protocols may also incorporate calcium as a physiological target rather than provide explicit replacement guidance; as such, further research could also benefit from assessing precision trauma care, with guided treatments based on the patient's calcium level.

- The limitations section requires expansion to address the methodological concerns raised above.
  - **Thank you. We believe we have addressed this throughout, but we hugely value any additional feedback that can be provided to further improve the quality of this study.**

- The conclusion states "considerable variation amongst EDs regarding the role" — it is unclear whether this refers to variation in ED protocols or in ED physician opinion on when to replace Ca. Please clarify.
  - **Thank you. The conclusion of the abstract now reads: 'There is considerable variation among EDs regarding ionised calcium measurement and calcium replacement both before blood product transfusion and in major haemorrhage**

protocols. **Clinicians' opinions on calcium measurement and replacement also vary widely. More research is needed to reach a consensus.'**

What Is Already Known

- First statement: please clarify whether the data on Ca disturbances and link to mortality refers to adult or paediatric populations. "Disturbance" is vague — please specify (hypocalcaemia, hypercalcaemia, or both).
    - **Thank you. We have updated this to reflect that both ionised hypo- and hypercalcaemia are linked to mortality, adverse outcomes, and increased treatment requirements in the adult trauma population.**

- Third point regarding MHPs: the relevance to this paper is unclear in its current form. A more specific reference to Ca within MHP would improve clarity.
    - **Thank you. We have updated this to now read: 'There is a lack of research exploring practice variation in how major haemorrhage protocols integrate ionised calcium measurement and calcium replacement for paediatric trauma, and variations in practice are reflected as an established research priority.'**

What This Study Adds

- The statement that all EDs can measure Ca assumes lab testing — clarify if point-of-care testing (POCT) is intended.
    - **Thank you. Throughout the article, we have moved away from point-of-care testing (POCT) when discussing the results of this study and instead emphasised that ionised calcium is measured on a blood gas.**
    - **Point-of-care testing remains part of the discussion in the wider context of prehospital emergency medicine.**

Future Research

- The reference to MHP should be more explicitly linked to Ca to improve relevance and clarity.

- **Thank you. This has been updated accordingly to read: ‘This underscores the need for further research into optimal thresholds, timing, and integration of calcium into major haemorrhage protocols, as well as consensus on what defines clinically significant hypocalcaemia before blood product transfusion’**
